# *Serratia symbiotica* Enhances Fatty Acid Metabolism of Pea Aphid to Promote Host Development

**DOI:** 10.3390/ijms22115951

**Published:** 2021-05-31

**Authors:** Xiaofei Zhou, Xiaoyu Ling, Huijuan Guo, Keyan Zhu-Salzman, Feng Ge, Yucheng Sun

**Affiliations:** 1 State Key Laboratory of Integrated Management of Pest Insects and Rodents, Institute of Zoology, Chinese Academy of Sciences, Beijing 100101, China; zhouxiaofei@ioz.ac.cn (X.Z.); lingxiaoyu@ioz.ac.cn (X.L.); guohj@ioz.ac.cn (H.G.); 2 CAS Center for Excellence in Biotic Interactions, University of Chinese Academy of Sciences, Beijing 100049, China; 3 Department of Entomology, Texas A&M University, College Station, TX 77843, USA; ksalzman@tamu.edu

**Keywords:** *Acyrthosiphon pisum*, development, endosymbiont, fatty acid, *Serratia symbiotica*

## Abstract

Bacterial symbionts associated with insects are often involved in host development and ecological adaptation. *Serratia symbiotica*, a common facultative endosymbiont harbored in pea aphids, improves host fitness and heat tolerance, but studies concerning the nutritional metabolism and impact on the aphid host associated with carrying *Serratia* are limited. In the current study, we showed that *Serratia*-infected aphids had a shorter nymphal developmental time and higher body weight than *Serratia*-free aphids when fed on detached leaves. Genes connecting to fatty acid biosynthesis and elongation were up-regulated in *Serratia*-infected aphids. Specifically, elevated expression of fatty acid synthase 1 (*FASN1*) and diacylglycerol-o-acyltransferase 2 (*DGAT2*) could result in accumulation of myristic acid, palmitic acid, linoleic acid, and arachidic acid in fat bodies. Impairing fatty acid synthesis in *Serratia*-infected pea aphids either by a pharmacological inhibitor or through silencing *FASN1* and *DGAT2* expression prolonged the nymphal growth period and decreased the aphid body weight. Conversely, supplementation of myristic acid (C14:0) to these aphids restored their normal development and weight gain. Our results indicated that *Serratia* promoted development and growth of its aphid host through enhancing fatty acid biosynthesis. Our discovery has shed more light on nutritional effects underlying the symbiosis between aphids and facultative endosymbionts.

## 1. Introduction

Insects are a highly successful group of animals, and some of them are able to utilize a wide range of nutrient-unbalanced food resources from the plant phloem sap to animal blood [1,2]. Heritable microbial symbionts are reported to shape insect adaption to diverse feeding habits [3,4]. The pea aphid *Acyrthosiphon pisum* usually hosts one obligate symbiont *Buchnera* and several facultative symbionts [5,6]. *Buchnera* offers essential amino acids to aphids, crucial for their survival on nutrition-unbalanced phloem sap [7]. By contrast, the facultative symbionts are usually unnecessary for host survival and reproduction. Instead, they improve the fitness and adaption of the aphid to environments [8].

The benefit of infection with facultative symbionts is known to provide hosts with protection against parasitoids and entomopathogenic fungi [9,10] and heat stress [11]. Maintenance of symbiosis, however, could be very costly in the absence of the stress. For example, *Hamiltonella defensa* strongly protects the pea aphid against a parasitoid, but causes a shorter lifespan and lower rate of reproduction in the absence of the natural enemy [12]. The physiological cost of carrying facultative symbionts may result from their competition for essential nutrients with the obligate symbionts or aphid hosts [13,14]. Interestingly, in some cases, the strength of protection is negatively correlated with the cost imposed on the host, suggesting that some facultative symbionts could provide a direct nutritional benefit to aphids in addition to the previously recognized protection [15]. In fact, the whole-genome sequencing of endosymbionts in sap-feeding insects indicated that nutrient-synthetic genes of facultative symbionts are horizontally transferred to insect hosts, which may enhance insect fitness [16,17]. Facultative symbionts can alter nutrient distribution in the host by modulating metabolic pathways to decrease glucose levels but increase fatty acid and amino acid syntheses [18,19].

In the mutualistic symbiosis, endosymbiont bacteria offer insect hosts essential amino acids that are deficient in the plant phloem sap. In turn, they receive carbohydrates or non-essential amino acids from insect hosts [20,21]. Fatty acids are also major carbon sources to maintain the symbiotic association between hosts and symbiotic bacteria, especially in flies with limited nutrition [14]. It has been shown that unsterilized aphids can be raised for several generations on lipid-deficient artificial diets, and, likewise, sterile aphids supplied with fatty acids can accelerate the development and produce fertile offspring, suggesting that fatty acids needed by aphids may be provided by endosymbionts [22,23]. However, direct evidence showing bacterial symbiont-regulated fatty acid metabolism in aphid hosts is still missing.

The facultative symbiont *Serratia symbiotic* harbored in pea aphids is reported to enhance host heat tolerance, provide protection against parasitoids, and suppress plant defenses [10,24,25,26]. *Serratia*-infected aphids avoid triggering calcium sparks and thus evade plant defenses. Such aphids also display better phloem feeding, presumably by allowing aphids to ingest more nutrients or directly modify nutrient metabolism. A pea aphid population collected from the alfalfa field has a *Serratia* infection rate as high as 85% without any obvious physiological cost, indicating that *Serratia* infection is beneficial to aphids [26]. To test this hypothesis experimentally, we established *Serratia*-infected and *Serratia*-free aphid lines originating from a single female and compared the effect of *Serratia* on the aphid host’s development and fatty acid synthesis.

## 2. Results

### 2.1. Serratia Infection Improved Growth and Development of Pea Aphids

**Figure 1:**
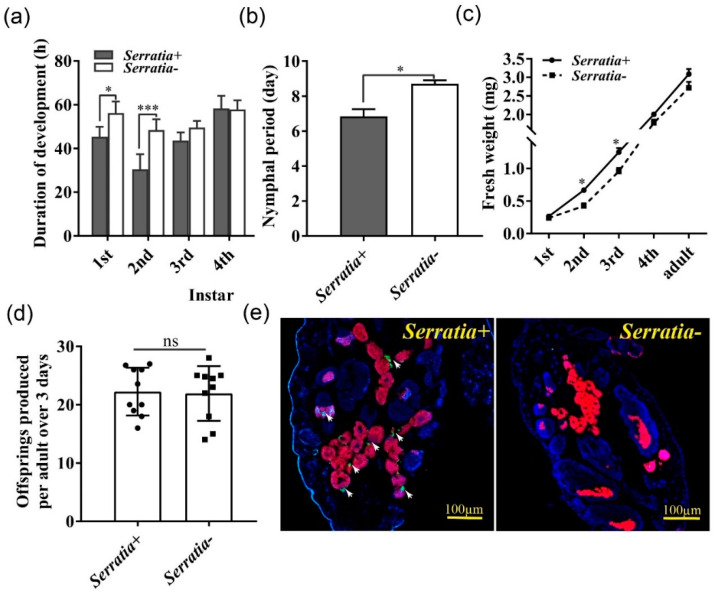
*Serratia* facilitated growth and development of pea aphids. Duration of (**a**) each, and (**b**) total nymphal developmental stage (*n* = 30). (**c**) Fresh weight (*n* = 30). (**d**) Fecundity (*n* = 10). (**e**) DNA-FISH was used to detect *Buchnera* and *Serratia* within aphids. Aphid DNA was stained with DAPI (blue). *Buchnera* DNA was hybridized with Cy5-labelled DNA probe (red) and *Serratia* DNA was hybridized with Cy3-labelled DNA probe (green). *Serratia*+: *Serratia*-infected aphid, *Serratia*-: *Serratia*-free aphid. The scale bar = 100 μm. Data shown are means ± SE. * indicated significant differences based on the Student’s t test at *p* < 0.05 (* *p* < 0.05, *** *p* < 0.001), while ns indicated no significant difference.

To determine the effect of *Serratia* infection on the performance of pea aphids, developmental time, body weight, and fecundity were compared between *Serratia*-free and *Serratia*-infected aphids when fed on detached broad bean leaves. *Serratia*-infected aphids had significantly shorter first and second instar nymphal duration and a shorter total nymphal period than *Serratia*-free aphids (Figure 1a,b). Consistently, infected second and thirrd instar nymphs had a higher body weight than *Serratia*-free aphids (Figure 1c). By contrast, *Serratia* infection did not significantly affect offspring numbers of pea aphids (Figure 1d). We applied fluorescence in situ hybridization to determine the locations of *Serratia* and *Buchnera* within pea aphids. As expected, *Buchnera* was detected in the bacteriocytes of both *Serratia*-free and -infected aphids, whereas *Serratia* appeared nearby bacteriocytes only in *Serratia*-infected aphids (Figure 1e).

### 2.2. Serratia Promoted Fatty Acid Synthesis and Lipid Accumulation in the Fat Body of Pea Aphid

**Figure 2:**
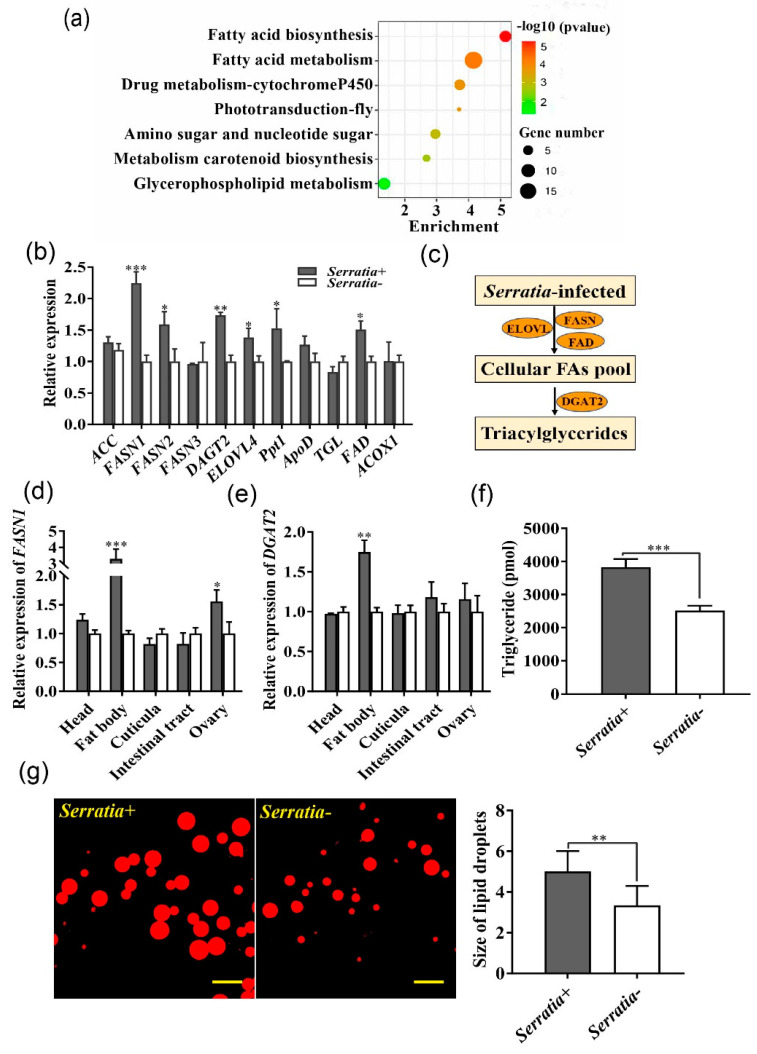
*Serratia* up-regulated fatty acid metabolism in pea aphids. (**a**) KEGG enrichment analysis of DEGs in the *Serratia*-infected and *Serratia*-free aphids. Bubble sizes represent DEG numbers in the pathway. (**b**) The relative expression of 11 high abundance genes associated with fatty acid metabolism in *Serratia*-infected (*Serratia*+) transcriptome when compared with *Serratia*-free (*Serratia*-) aphids. *ACC*: acetyl-CoA carboxylase; *FASN*: fatty acid synthase; DGAT2: diacylglycerol O-acyltransferase2; *ELOVL4*: elongation of very long chain fatty acids protein 4; *Ppt1*: palmitoyl-protein thioesterase1; *ApoD*: apolipoprotein D; *TGL*: triacylglycerol lipase-like; *FAD*: fatty acid desaturase-like; *ACOX1*: probable peroxisomal acyl-coenzyme A oxidase 1. (**c**) The schematic diagram highlighting up-regulated lipid synthetic genes in *Serratia*-infected aphids. (**d**,**e**) Relative expression of *FASN1* and *DGAT2* in different tissues of *Serratia*-infected aphids. (**f**) Triglyceride concentration in *Serratia*+ and *Serratia*- fat bodies (*n* = 30). (**g**) Lipid droplets in fat bodies of *Serratia*- and *Serratia+* aphids using Nile red staining; The scale bar = 50 μm. Values in plot bar are means ± SE. Data were compared with controls using the Student *t* test: * *p* < 0.05; ** *p* < 0.01, *** *p* < 0.001.

To assess the effect of *Serratia* infection on transcription of pea aphids, RNAseq was conducted using 2nd–3rd instar nymphs from *Serratia*-free and *Serratia*-infected aphids. Transcriptome analysis showed that 121 genes were significantly up-regulated in *Serratia*-infected aphids (Table S4), and 200 genes were significantly down-regulated relative to *Serratia*-free aphids (Table S5). KEGG analysis revealed that these differentially expressed genes (DEGs) were enriched in seven pathways including those for fatty acid biosynthesis and metabolism (Figure 2a, Table S3). Based on RNAseq data, 11 genes related to fatty acid metabolism were of high abundance, and six of them were confirmed by qPCR (Figure 2b). Of which, *FASN1* and *DGAT2*, necessary for adipogenesis, displayed the greatest differences in gene expression (Figure 2c). Furthermore, we determined the relative expression of these two genes in different aphids’ tissues, and found that *FASN1* and *DGAT2* were almost four-fold and three-fold higher, respectively, in fat bodies of *Serratia*-infected aphids than those of *Serratia-free* aphids. In contrast, expression was comparable in other tissues with the exception of *FASN1* in the ovary (Figure 2d,e). Since *Serratia* infection increased lipogenesis by up-regulating *FASN1* and *DGAT2*, we then quantified the lipid contents in the aphid fat body, and showed that *Serratia*-infected aphids had more triacylglycerols and larger lipid droplets than *Serratia*-free aphids (Figure 2f,g).

### 2.3. More Fatty Acids Accumulated in Serratia-Infected Aphids, Which Facilitated Aphid Growth

**Figure 3:**
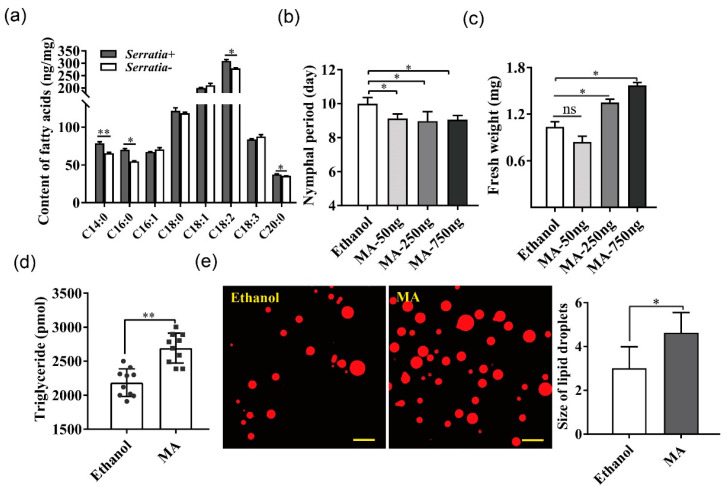
Effects of exogenous fatty acid application on *Serratia*-free aphids. (**a**) GC-MS was performed to analyze free fatty acid contents in *Serratia*-infected and *Serratia*-free aphids, respectively. Only the top 8 were shown because they represented more than 95% contents of measured fatty acids. C14:0 myristic acid (MA); C16:0 palmitic acid (PA); C16:1 palmitoleic acid (PMA); C18:0 stearic acid (SA); C18:1 oleic acid (OA); C18:2 linoleic acid (LA); C18:3 linolenic acid (α-LA); C20:0 arachidonic acid (AA). (**b**,**c**) Nymphal period and body weight of pea aphids treated with different amounts of MA. (**d**) The triglyceride content in *Serratia*-free aphids injected with 250 ng MA. (**e**) Lipid droplets in fat body of *Serratia*-free aphids fed with 250 ng MA using Nile red staining. Scale bar = 50 μm. Values in plot bars are means ± SE. Data were compared with controls using the Student’s *t* test: * *p* < 0.05; ** *p* < 0.01; while *ns* indicated no significant difference.

A total of 26 fatty acids, all long-chain, were identified in pea aphids using GC-MS, including nine saturated, eight monounsaturated, and nine polyunsaturated fatty acids (Table S2). Eight of the 26 fatty acids accounted for more than 95% of the total fatty acids, among which the contents of C14:0 (myristic acid), C16:0 (palmitic acid), C18:2 (linoleic acid), and C20:0 (arachidonic acid) were significantly higher in *Serratia*-infected than *Serratia*-free aphids (Figure 3a, Table S2).

Since myristic acid was the major fatty acid of triacylglycerol fraction in pea aphids and was highly accumulated in *Serratia*-infected aphids, we then determined the effect of exogenous myristic acid on *Serratia*-free aphids using micro-injection. The injection of 250 and 750 ng myristic acid not only shortened the nymphal period but also increased the body weight of aphids, whereas the injection of 50 ng myristic acid only shortened development time (Figure 3b,c). Meanwhile, we detected that the triacylglycerol content (Figure 3d) and lipid droplet size (Figure 3e) in *Serratia*-free aphids injected with 250 ng myristic acid were also significantly higher than the ethanol control two days after injection. None of the myristic acid doses used in this study affected the fecundity of *Serratia*-infected aphids (Figure S3).

### 2.4. Silencing FASN1 and DGAT2 Suppressed Lipogenesis and Reduced Fitness of Serratia-Infected Aphids

**Figure 4:**
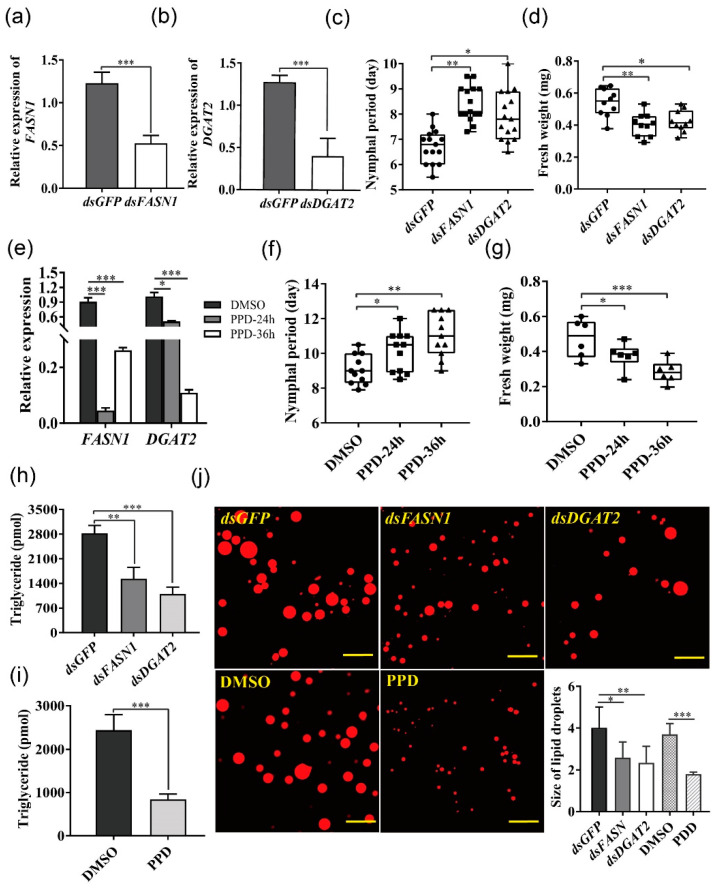
*FASN1* or *DGAT2* silencing or PPD treatment delayed growth and development of *Serratia*-infected aphids. (**a**) *FASN1* and (**b**) *DGAT2* expression in *Serratia*-infected aphids after feeding with ds*FASN1* and ds*DGAT2*, respectively (*n* = 6). (**c**,**d**) Effects of silencing *FASN1* and *DGAT2* on the nymphal period and the body weight, respectively (*n* = 10–15). Data were compared with those of nymphs fed with ds*GFP*. (**e**) *FASN1* and *DGAT2* expression levels of *Serratia*-infected aphids fed with PPD within 36 h (*n* = 6). (**f**,**g**) Effects of pharmacological inhibitor PPD on the nymphal period and body weight, respectively (*n* = 6–11). Data were compared with those of nymphs fed with DMSO. (**h**,**i**) Triglyceride contents were detected in *Serratia*-infected aphids fed with (**h**) ds*FASN1*/ds*DGAT2* and (**i**) PPD (*n* = 8). (**j**) Lipid droplets changes in fat body of *Serratia*-infected aphids fed with dsRNA or PPD using Nile red staining. Scale bar = 50 μm, *n* > 5. Box plots represent the median (bold black line), quartiles (boxes), as well as the minimum and maximum (whiskers). Values in bar plots represented means ± SE (* *p* < 0.05; ** *p* < 0.01; *** *p* < 0.001); ns, non-significant difference.

ds*FASN1*- and ds*DGTA2*-feeding significantly reduced gene expression (Figure 4a,b) and triacylglycerol content (Figure 4h,j) 24 h after injection in *Serratia*-infected aphids. Silencing *FASN1* and *DGTA2* prolonged the larval duration and decreased the body weight of *Serratia*-infected aphids (Figure 4c,d), but did not affect reproduction (Figure S2a). Furthermore, we used the pharmacological inhibitor, pseudoprotodioscin (PPD), to interfere with the lipid synthesis. It has been shown that PPD inhibits sterol-regulatory element-binding protein1/2 (REBP1/2) and miRNA 33a/b levels to reduce the synthesis of cholesterol and triglycerides without affecting food consumption [27]. Expression levels of *FASN1* and *DGAT2* were significantly decreased by PPD ingestion after 24 h compared with the control (Figure 4e), leading to a reduced triacylglycerol content (Figure 4i,j). Similarly, PPD feeding for 24 h significantly prolonged the nymphal period, decreased the aphid weight (Figure 4f,g), as well as affected fecundity after 36 h (Figure S2b).

### 2.5. Supplementation with Myristic Acid Rescued the Fitness of Serratia-Infected Aphids Impaired by RNAi or PPD

**Figure 5:**
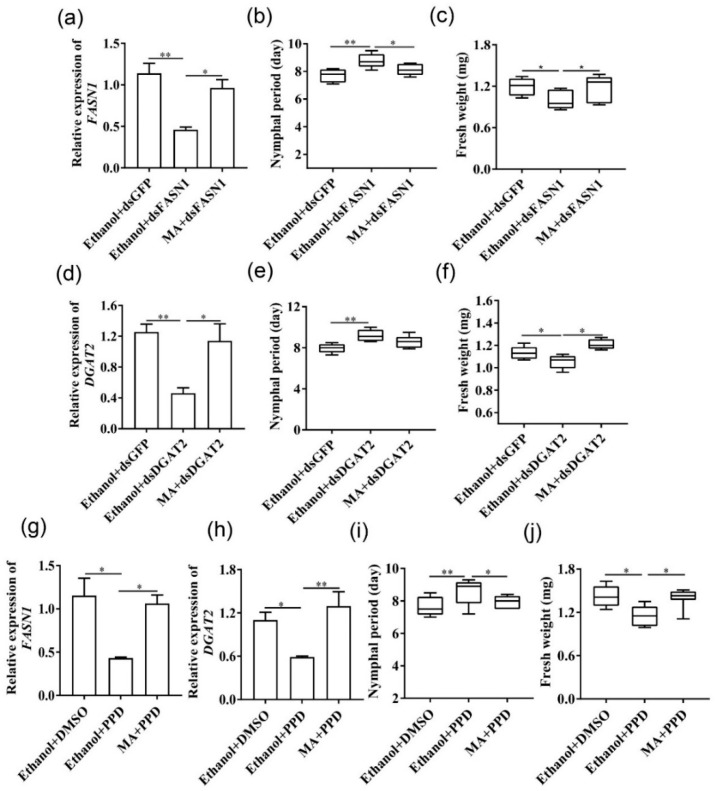
Supplementation with myristic acid resumed fitness of *Serratia*-infected aphids from fatty acid synthesis impairment. *FASN1* (**a**–**c**) and *DGAT2* (**d**–**f**) relative expression, nymphal period, and fresh weight of ds*GFP* vs. ds*FASN* or ds*DGAT2* aphids that were injected with MA or ethanol 10 h later from RNAi treatment (*n* = 30). (**g**–**j**) *FASN1* and *DGAT2* relative expression, nymphal period, and fresh weight of DMSO- vs. PPD-treated aphids that were injected with MA or ethanol 10 h later from RNAi treatment (*n* = 21). Box plots represent the median (bold black line), quartiles (boxes), as well as the minimum and maximum (whiskers). Values are means ± SE. Statistical significance was determined by the Student’s *t* test: * *p* < 0.05; ** *p* < 0.01; while ns indicated no significant difference.

ds*FASN1* treatment knocked down *FASN1* expression in aphids by 59.6%, increased the nymphal duration, and reduced body weight. Myristic acid (C14:0) injection to aphids that were fed with ds*FASN1*, however, mostly restored the *FASN1* transcript level, normal nymphal duration, and body weight (Figure 5a–c). The same is true for *DGAT2*-silenced aphids (Figure 5d–f). As expected, supplementation of myristic acid significantly also resumed expression of *FASN1* and *DGAT2* in PPD-treated aphids, shortened the nymphal duration, and led to body weight gains (Figure 5g–j). These results indicated that exogenous supplementation of myristic acid could rescue aphids from reduced fitness.

## 3. Discussion

The facultative endosymbionts are often beneficial to aphid hosts when challenged by environmental stresses, but unfavorably affect host fitness when the stress is relieved [15]. Widespread evidence has shown that aphid facultative endosymbionts, such as *H. defensa*, *Spiroplasma*, and *Rickettsia*, decrease growth, lifespan, fecundity, and/or body weight of aphid hosts [28,29,30]. *Serratia* in pea aphids, however, may represent an exception since it facilitated the growth and development of aphid host by enhancing fatty acid synthesis and increasing triacylglycerol storage. Similar to our findings, *Serratia* in the cedar aphid *Cinara cedri* provides essential amino acids to aphids, which accelerates host growth [31]. Similar to other bacteria symbionts, *Serratia* could utilize a variety of carbon sources from aphid hosts [32]. We showed that *Serratia* infection facilitated the accumulation of fatty acids by up-regulating the expression of *FASN1* and *DGAT2* in the lipogenesis pathway. It suggested that the outcome of physiological cost caused by endosymbionts was most likely determined by the utilization and allocation of nutritional resources between the facultative endosymbiont and its aphid host.

Insect endosymbionts may compete with each other for resources and space within the same host [33]. For example, injected *Serratia* bacteria are dispersed in the hemolymph of the pea aphid, and these *Serratia* cells frequently invade the primary bacteriocytes, leading to decreased *Buchnera* density [13]. Surprisingly, *Serratia* infection of *Buchnera*-free aphids indeed can prevent the survival rate and fecundity from dropping, suggesting that *Serratia* could, at least in part, supply the aphid host with essential amino acids and other nutrients as *Buchnera* does [13]. Our *Serratia*-infected aphid clone was originally collected in nature instead of artificially generated by hemolymph injection. *Serratia* is located largely in sheath cells of the aphid host, spatially separated from primary bacteriocytes. This could explain our finding that *Serratia* infection had little effect on *Bucherna* abundance in our aphid clone. Logically, it is less likely for *Buchnera* to be responsible for the positive effect of carrying *Serratia* on the fitness of the pea aphid (Figure S1). The contrasting effects of *Serratia* infection on *Buchnera* in pea aphids are possibly due to how *Serratia*-infected clones were generated, i.e., a natural clone that has already hosted both symbionts versus a newly infected clone by hemolymph injection. In the natural *Serratia*-infected clone, we speculated that *Serratia* improved aphid fitness by directly promoting its nutritional metabolism rather than indirectly influencing *Buchnera*.

Since fatty acids are crucial for insect growth, reproduction, and immunity, the metabolic pathways and enzymes involved are evolutionarily conserved in insects [34]. Our transcriptomic data showed that key genes associated with fatty acid synthetic pathways were up-regulated in *Serratia*-infected aphids, similar to the results obtained from *Hylyphantes graminicola* co-infected with *Wolbachia* and *Cardinium* [19]. Consistently, higher concentrations of C14:0, C16:0, C18:3, and C20:0 were also observed in *Serratia*-infected aphids than those of *Serratia*-free aphids, which likely led to a shorter development time and higher body weight in *Serratia*-infected aphids. This agrees with the consensus that the accumulation of lipids and fatty acids is typically associated with a better fitness in insects [35]. Furthermore, unlike *Wolbachia* that lacks pathways for fatty acid synthesis, *Serratia* is able to synthesize some fatty acids, especially for short-chain fatty acid acetate [32,36]. It was speculated that acetate could be released into the host hemolymph and delivered into fat body cells as substrates for fatty acid synthesis. It has been extensively studied that some short-chain fatty acids, including acetate, produced by intestinal symbiotic bacteria can regulate the fatty acids synthesis of the mammalian host [37], but more details need to be determined about how facultative endosymbionts synthesize short-chain fatty acids in aphids. Furthermore, we also found that lipogenesis genes were highly expressed in the fat body, suggesting that peripheral sheath cells of the bacteriomes where *Serratia* is located may interact with the scattered fat body cells in the aphid hemolymph. The fat body is a nutrition-rich environment for endosymbionts in many insects [38]. It has been shown that supplementing cholesterol in the diet led to increases in *Wolabchia* titer in the mosquito *Aedes albopictus* [39]. By contrast, suppression of lipogenesis in the pea aphid had little effect on the titer of *Serratia*, suggesting that lipids may not be necessary for the proliferation of *Serratia*, possibly because of its fatty acid synthetic ability in [32,40]. Since the fat body is the central repository of nutrients and energy, the enhanced lipid storage in lipid droplets can also better meet the aphid’s energy demands for growth, in addition to enhanced tolerance to the heat or cold stresses [41].

Silencing *FASN1* and *DGAT2* suppressed lipogenesis and fitness of *Serratia*-infected aphids, which could then be rescued by exogenous myristic acid supplementation in both *Serratia*-free and *Serratia*-infected aphids. Perhaps, myristic acid promoted the lipid synthesis necessary for aphid growth and development. In insects, dietary fatty acids are absorbed by midgut enterocytes to synthesize DAG that is then transferred to the fat body and converted to TAG by DAGT1/2 [34,42]. Likewise, the current study showed that myristic acid supplementation up-regulated *DGAT2* expression in the fat body and eventually led to the synthesis of TAG and lipid droplet in pea aphids. Furthermore, FASN catalyzed the formation of palmitate acid (C16:0) via condensation of malonyl-CoA and acetyl-CoA, which could increase the lipid storage in the pea aphid by de novo fatty acid synthesis [43]. It has been reasonably speculated that supplementation of myristic acid (C14:0) in pea aphids increased the substrates for *FASN1* to produce palmitic acid [44]. These results suggested that exogenous myristic acid could up-regulate expression of *DAGT2* and *FASN1* to synthesize lipids and facilitate aphid growth.

Growing evidence suggested that *Serratia* has evolved into a co-obligate symbiont with *Buchnera.* As an integrated unit, they function collectively in many aphid species, such as the *Periphyllus* genus, to synthesize and express essential nutrients-provisioning genes that are lost in *Buchnera* [45]. Co-obligate symbionts are located within the same bacteriome, and removal of *Serratia* had the most dramatic effect on aphid survival and reproduction. In contrast, *Serratia* in *A. pisum* localized in sheath cells surrounding the *Buchnera*-containing bacteriocytes maintained a facultative symbiosis with the aphid host. They had little effect on pea aphid survival and lifetime fecundity, but improved the fitness of hosts. Indeed, many maternally transmitted bacterial symbionts provide some nutritional supplements to their hosts that enable them to increase transmission frequency [46], explaining the prevalence of *Serratia* in insect populations. Overall, the main finding of this study showed that *Serratia* infection significantly up-regulated fatty acid biosynthesis, rendering more lipid accumulation in aphids, which directly enhanced the fitness of the aphid host regardless of plant defenses. It provided deep understanding on the nutritional basis for maintaining the symbiosis of the aphid host and its facultative endosymbiont.

## 4. Materials and Methods

### 4.1. Aphid Rearing

The pea aphid (*A. pisum*) clone used in this experiment was originally collected from the *Medicago sativa* field at Yinchuan, Ningxia, China in 2015. *Serratia*-infected aphids were parthenogenetic descendants from a single isolated female. We established *Serratia*-free aphids by injecting ampicillin into the same *Serratia*-infected clone [26]. All aphids were reared on broad bean *Vicia fabae* at 20 ± 1 °C, 70–80% relative humidity and a photoperiod of 16:8 (L:D) in photoclimate chambers (Safe PRX-450C, Ningbo, China). The aphids had been reared in the laboratory for three years at the start of this study. During the maintenance of these strains, the infection status was checked by diagnostic PCR every month.

### 4.2. Comparison of Life History Parameters

Newly born nymphs (<10 h) randomly selected from *Serratia*-free and *Serratia*-infected aphids were placed in 60-mm-diameter petri dish with a detached broad bean leaf to record the nymphal developmental time from birth to adult. All petri dishes were placed in a growth chamber (Safe PRX-450C, Ningbo, China) with a 14 h light (22 °C) and 10 h dark (20 °C) photoperiod. Each instar stage of nymphal duration was monitored every 8 h. The body weight of the aphid was determined using an automatic electro-balance at the early stages of every instar and the adult. Fecundity was indicated as the number of offspring produced within three days from the beginning of reproduction. One aphid from one dish was considered as a replicate for determinations of developmental time and fecundity, and 30 dishes in total were used for each treatment. Five 1st–2nd instar nymphs were randomly selected as a replicate to measure the body weight, and six replicates were used for each treatment. Fresh leaves were provided every day to standardize the nutritional effect of diet on all aphids.

### 4.3. Fluorescence In Situ Hybridization (FISH)

FISH was employed to localize *Serratia* and *Buchnera* performing as described previously [13]. The fluorescent probe ApisP2a-Cy5 (5′-Cy5-CCTCTTTTGGGTAGATCC-3′), targeted the 16S rRNA of the primary symbiont *Buchnera*, and the probe PASSisR-Cy3 (5′-Cy3-CCCGACTTTATC GCTGGC-3′) specifically targeted *Serratia* 16S rRNA. Nuclei of the host cell were counterstained with 4′,6-diamino-2-phenylindole (DAPI). To confirm the specific detection, no probe and RNase digestion were conducted as controls.

### 4.4. RNA Extraction and Reverse Transcription Quantitative PCR

The whole body or different tissues (fat body, head, ovary, cuticula, intestinal tract) from ten 2nd–3rd instar nymphs in each sample were homogenized using a motor-driven pellet pestle mixer and were lysed by the TRIzol reagent (Invitrogen, Carlsbad, CA, USA). RNA concentration was evaluated using the NanoDrop 1000 spectrophotometer (Thermo Scientific, Wilmington, DE, USA), and then 1 μg RNA was used to synthesize the first-strand cDNA (20 μL) with the FastQuant RT Kit (Tiangen, Beijing, China) according to the manufacturer’s protocol. Four biological replicates were conducted for each treatment, and each biological replicate contained three technical repeats. To detect gene expression, qPCR was performed in a 20 μL reaction volume containing 10 μL of the 2×SYBR Premix Ex Taq (Tiangen, Beijing, China) master mix, 7 μL water, 1 μL of cDNA template, and 1 μL of gene-specific primers (Table S1). Reactions were carried out on the Mx 3500P detection system (Stratagene, La Jolla, CA, USA): 15 min at 95 °C, followed by 40 cycles of 10 s at 95 °C, 20 s at 56 °C, and 30 s at 72 °C. Elongation factor 1α (EF1-α) was used as the reference gene for normalization of gene expression. Data were collected from three independent biological replicates and at least six technical replicates and analyzed via the 2^−^^ΔΔ^^CT^ method.

### 4.5. Determination of Free Fatty Acid Levels

The fat bodies dissected from ten 2nd–3rd instar nymphs were frozen in liquid nitrogen and ground in 1 mL of a 2% H_2_SO_4_/98% methanol solution (5 μL 200 ug/mL C19:0 as internal reference). Samples were sealed with a cap and incubated at 80 °C for 1 h. After the addition of 0.3 ml of hexane and 1.5 ml of H_2_O, the fatty acid methyl esters were extracted into the hexane layer by shaking and then were centrifuged at 5000× *g* for 10 min. Samples of the organic phase were carried out by means of GC-MS on an Agilent Technologies 6890N GC-5973N mass selective detector. The GC was equipped with a HP-5MS column (60 mm × 0.25 mm; film thickness 0.25 μm) (J&W Scientific, Folsom, CA, USA) and carried out following the procedure described previously [47]. Compounds were identified by comparing their retention time with those of authentic reference compounds and comparing the spectra with that of the mass spectral library NIST02 (Rev. D.04.00; Agilent Technologies, Palo Alto, CA, USA).

### 4.6. Triacylglycerol Measurements

The total triacylglycerols (TAGs) level was measured using the Picoprobe Triglyceride Quantification Assay Kit (Abcam, Cambridge, UK). Briefly, fat bodies dissected from 30 3rd instar nymphs were homogenized in a 100 μL ice cold triglyceride assay buffer and incubated on ice for 10 min. Following a centrifugation, the supernatant was transferred into 96-well plates, incubated with the reaction mix at 37 °C for 30 min and placed in a microplate reader SpectraMax Plus384 (Molecular Devices, Silicon Valley, CA, USA,) with an excitation wavelength of 535 nm and an emission wavelength of 587 nm. Four independent biological replicates and four technical replicates were performed for every treatment.

### 4.7. Nile red Staining

Lipid was visualized by staining the second instar nymph fat bodies with Nile Red. Fat bodies were dissected and washed 2–3 times with a 1×PBS buffer (pH 7.4), and the adherent tissues were carefully removed with forceps under a stereomicroscope (SMZ745, Nikon, Tokyo, Japan ). The dissected fat bodies were fixed with 4% paraformaldehyde on a glass slide for 2 h at room temperature and then washed with PBS three times (3 min × 5 min). For lipid staining, fat bodies were submerged in Nile red solution (MCE, Monmouth Junction, NJ, USA) at a final working concentration of 10 μg/ml in an acetone/water (1:9) mixture and visualized using a fluorescent microscope (LSM 710, Zeiss, Carl, Germany) at Ex543/Em626 nm. The mean size of lipid droplets from each sample was analyzed by Image J software (V1.8.0, 2017).

### 4.8. Inhibition of Fatty Acid Synthesis in Aphids

To silence genes by oral ingestion of dsRNA, dsRNAs specific to aphid *FASN1*, *DGAT2*, and *GFP* were synthesized using the T7 RiboMAX Express RNAi system kit (Promega, Madison, WI, USA), following the manufacturer’s protocol. The primers used are listed in Table S1. ds*GFP* served as a negative control. dsRNA was delivered into the 1st-instar nymphs (2-day-old) by oral administration. Briefly, about 30 *Serratia*-infected nymphs were restrained in a petri dish (Φ3.5 cm) covered with two layers of parafilm filled with a 20% (wt/vol) sucrose diet containing dsRNA at the concentration of 250 ng/μL. After 24 h feeding, five aphids were collected for assessment of RNAi efficacy by qPCR. Once significant interference efficiency was observed, the rest of the treated aphids were transferred to detached leaves on 1.5% (*w*/*v*) agar in a petri dish (Φ9.5 cm) until they became adult aphids. They were then collected for fitness measurement, triacylglycerol detection, and Nile red staining.

Pseudoprotodioscin (PPD) was used to inhibit gene expression related to the synthesis of triglycerides [27]. The 1st instar nymphs were placed in the aforementioned feeding device containing 300 μL 20% sucrose solution mixed with 6 μg of stock PPD solution (10 mM, MCE, Monmouth Junction, NJ, USA), and the nymphs fed with the sucrose with 5% DMSO represented the control. Some of the treated nymphs were collected at 12 h, 24 h, and 36 h after ingesting for further analysis of gene expression, and for the determination of aphid performance. Four biological replicates were performed. At least 30 aphids were used for each treatment.

### 4.9. Exogenous Myristic Acid Supplementation

Using a microinjector (Drummond Scientific Company, Broomall, PA, USA), 50, 250, and 750 ng of the original myristic acid (MA) dissolved in 50 nL ethanol were infected into hemolymph from dorsal abdomens in the 1st instar *Serratia*-free aphid nymphs (2-day-old, n > 30). Meanwhile, an equal amount of ethanol was injected as the solvent control. For *Serratia*-infected aphids, delivery of PPD and dsRNA was performed as described above. Twelve hours later, each aphid was injected with 50 nL MA (250 ng) or ethanol. All aphids were collected for weighing, nymphal period, and fecundity observation. Four biological replicates were conducted for each concentration.

### 4.10. Data Analysis

Data were shown as the means of at least four biological replicates with standard error (SE). Aphids that died during the experiments were discarded. The significance of differences was analyzed by using the Student’s *t* test for paired comparisons on GraphPad Prism 7.0 (GraphPad Software, San Diego, CA, USA). Asterisks represent statistical significance between groups (* *p* < 0.05, ** *p* < 0.01, *** *p* < 0.001).

## Data Availability

Data is contained within the article or Supplementary Material.

## Supplementary Materials

The following are available online at https://www.mdpi.com/article/10.3390/ijms22115951/s1. Figure S1: The relative abundance of *Buchnera* in nymph and adult of *Serratia*-infected and *Serratia*-free aphids, Figure S2: Application of 1st instar nymphs with RNAi (a) and PPD (b) and the effect on the fecundity of *Serratia*-infected aphids, Figure S3: Supplementation of different concentrations of myristic acid on 1st instar nymphs and the consequences for the fecundity of *Serratia*-free aphids, Figure S4: Effects of PPD ingestion within 12 h on the development of *Serratia*-infected aphids, Table S1: Primers used in this study, Table S2: Fatty acids detected in *Serratia*-infected and *Serratia*-free aphids, Table S3: Top 7 enrichment KEGG pathways in the DGEs, Table S4: Transcriptome: up-regulated mRNAs (*Serratia*-free aphids vs. *Serratia*-infected aphids), Table S5: Transcriptome: down-regulated mRNAs (*Serratia*-free aphids vs. *Serratia*-infected aphids)
