# Supplementary material for: Serratia symbiotica Enhances Fatty Acid Metabolism of Pea Aphid to Promote Host Development
Source: Int J Mol Sci. 2021 May 31;22(11):5951. doi: 10.3390/ijms22115951 (PMC8199403; doi:10.3390/ijms22115951)
Supplement: Supplementary file 1 [file ijms-22-05951-s001.zip › ijms-1226718-supplementary.pdf]

Supplementary Materials:

Supporting Online Material for

Serratia symbiotica enhances fatty acid metabolism of pea aphid to promote host development

Xiaofei Zhou[1,2], Xiaoyu Ling[1,2], Huijuan Guo[1,2], Keyan Zhu-Salzman[3], Feng Ge[1,2,*], and Yucheng Sun[1,2,*]

*** Corresponding authors:**

Tel: 8610 6480 7130; Fax: 8610 6480 7099; Email: gef@ioz.ac.cn (F. Ge); sunyc@ioz.ac.cn (Y. Sun)

Materials & Methods

Pea aphid transcriptome analysis

The new born 1st instar nymphs were transferred to a leaf on 1.5% (w/v) agar in a culture dish (Φ 9.0 cm) for 3 days. Total RNA was extracted from 30 2nd-3rd instar nymphs aphids for every replication and three replications per sample were sequenced. The clean data were obtained by removing low quality reads from the raw data with htseq-count software[1]. After quality control, the paired-end clean reads were aligned to the reference genome from NCBI website (https://www.ncbi.nlm.nih.gov/genome/?term=pea+aphid). FPKM value of each gene in each sample was calculated using Cufflinks. We identified differentially expressed genes with DEseq using per gene expected counts for each sample generated by FPKM. P value < 0.05 and $|\log_2(\text{FoldChange})| > 1$ was set as the threshold for significantly differential expression. KEGG pathway enrichment analyses of DEGs were respectively performed using R based on the hypergeometric distribution.

Quantitative PCR (qPCR)

DNA was extracted from different developmental stages of aphids with and without *Serratia* infection to determine whether *Serratia* infection in aphids influences the relative abundance of the primary symbiont *Buchnera*. qPCR was carried out in 20 μl volumes containing 10 μl of SYBR Green PCR Mix (Takara Bio, Shiga, Japan), 0.4 μl of 50× ROX Dye II, 1 μl of each primer, 1 μl of DNA and 6.6μl of sterile distilled water. Cycling conditions were 95℃ for 15min, then 40 cycles of 10 s at 95℃, 20 s at the annealing temperature at 55˚C, and 32 s at 72˚C. The relative abundance was normalized to the aphid elongation factor 1α gene (*EF1-*

α) and calculated using the $2^{-\Delta\Delta ct}$ method. The specific symbionts primers used in this study are listed in Table S1. Six biological replicates were performed.

Supplementary figures

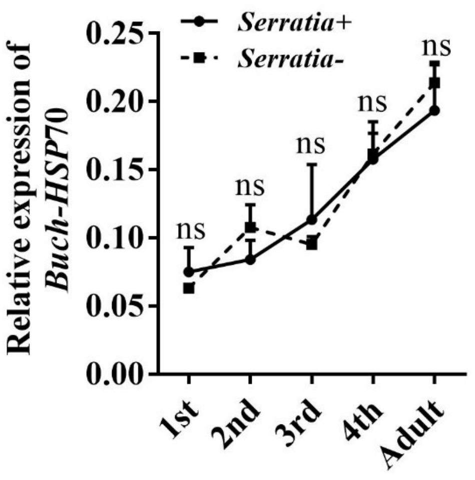

Figure S1. The relative abundance of *Buchnera* in nymph and adult of *Serratia*-infected and *Serratia*-free aphids. *Buch-HSP70* was specifically amplified the *HSP70* gene from *Buchnera*. Data of *HSP70* gene was normalized to the aphid *EF1-α* reference gene. Values are means ± SE. Statistical significances were determined by the Student's t test at $p < 0.05$ and ns indicated no significant difference.

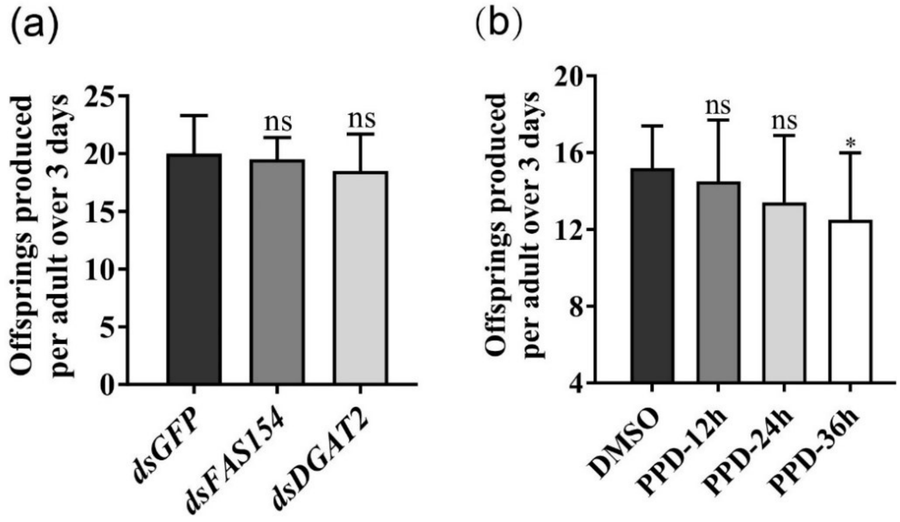

Figure S2. Application of 1st instar nymphs with RNAi (a) and PPD (b) and the effect on the fecundity of *Serratia*-infected aphids. Fecundity was indicated as the number of offspring produced within three days from the beginning of reproduction. Values were means ± SE. The asterisks indicated significant difference with the Student's t test at $p < 0.05$; ns represented non-significant difference.

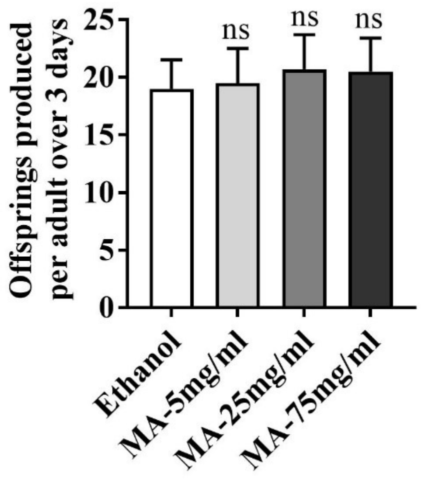

Figure S3. Supplementation of different concentrations of myristic acid on 1st instar nymphs and the consequences for the fecundity of *Serratia*-free aphids. Fecundity was indicated as the number of offspring produced within three days from the beginning of reproduction. Values were means ± SE. Statistical significances were determined by the Student's t test at $p < 0.05$ and ns indicated no significant difference.

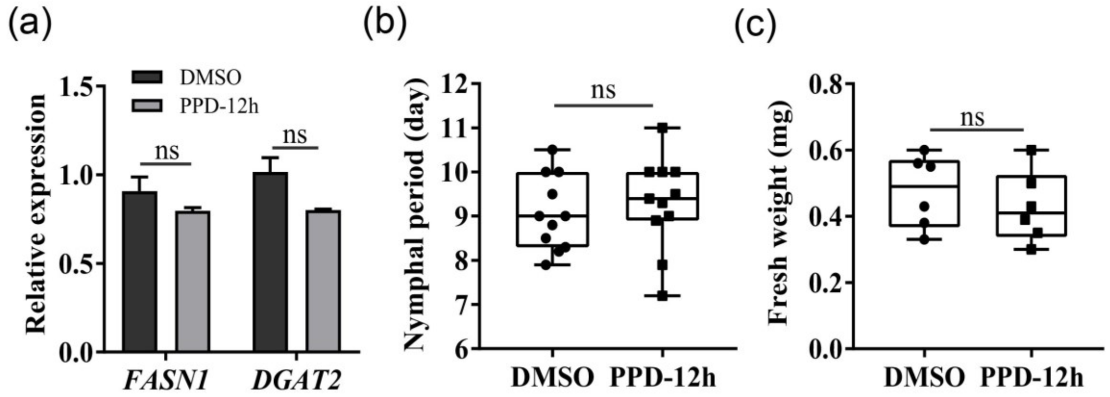

Figure S4. Effects of PPD ingestion within 12h on the development of *Serratia*-infected aphids. (a) *FASN1* and *DGAT2* expression levels of *Serratia*-infected aphids fed with PPD within 12h (n=6). (b-c) Effects of pharmacological inhibitor PPD on the nymphal period and body weight, respectively (n=6-11). Values were means ± SE. Statistical significances were determined by the Student's t test at $p < 0.05$ and ns indicated no significant difference.

Supplementary tables

Table S1. Primers used in this study

Primer name	Sequences (5'-3')	Length (bp)
For qPCR		
ApRF1	TGGCGGGTGATGTGAAG	120
ApRR1	CGGGATAGTGGTGTTTTTGG	
BuchHSP70F2	ATGGGTAAAATTATTGGTAATTG	130
BuchHSP70R2	ATAGCTTGACGTTTAGCAGG	
FASN1-F	GGGATCACTTTCCCGAGTGG	125
FASN1-R	TCGCCTACTTTAGTCCCCGT	
FASN2-F	GATGCAGTTGTGGTGCCTCT	154
FASN2-R	CGTTGGATTCTTCAGCGGTC	
FASN3-F	CACAAAGTTCAGCCGTCGTT	120
FASN3-R	GCGGATCTTGAATCCCCATAA	
ACC-F	TTCGGATGACGCGGATGAC	201
ACC-R	CCTTGAGACATACTAGGCGTGA	
DGAT2-F	GTTGCTGTTCTTATGGTAGGAGGT	118
DGAT2-R	ACGCCCCAGTTTGTAATGC	
ELOVLF4-F	TGATGGAAAACCGGGAACCA	111
ELOVLF4-R	TCCCGGGTGTGAAAAATACCT	
Ppt-F	TGGAATGGGCGATAGCTGTT	246
Ppt-R	GTGAGGAAAACTCAAGTAGCCC	
TGL-F	TCCAGACGCTTCGTTGAGTG	205
TGL-R	AGGCCCGTTGATTATCCAGT	
ApoD-F	CTAACACCGCACGACGACAA	234
ApoD-R	GACGGCTTTCGTGTAGTTGGT	
FAD-F	TGGTGAAGGCTGGCATAACT	197
FAD-R	CCAACTTCCATCTCCGTGCT	
ACOX1-F	CATCGTGGGATACACCCGTT	120
ACOX1-R	TCCGTTGTTCGCAGAGTTCA	
Ap*EF1-α*F	CTGATTGTGCCGTGCTTATTG	140
Ap*EF1-α*R	TATGGTGGTTCAGTAGAGTCC	
For dsRNA		
dsFANS1-F	TAATACGACTCACTATAGGGGCTTGTCCTGGGAAGATACT	609
dsFANS1-R	TAATACGACTCACTATAGGGCATAGAATAGGTCAGCGTGC	
dsGFP-F	TAATACGACTCACTATAGGGCACAAGTTCAGCGTGTCCG	400
dsGFP-R	TAATACGACTCACTATAGGGGTTCACCTTGATGCCGTTC	
dsDGAT2-F	TAATACGACTCACTATAGGGCCTACCGATGCCAACTACGA	388
dsDGAT2-R	TAATACGACTCACTATAGGGCACCGCCAGATTGAATAAGA	

Table S2. Fatty acids detected in *Serratia*-infected and *Serratia*-free aphids. Values are means ± SE. The asterisks indicated significant difference with the Student's t test at $p < 0.05$

Precursor Ion	Retention	Content (ng/mg)		
Name	Time	*Serratia+*	*Serratia-*	P
FA(14:0)	7.44	78.61	65.52	0.001**
FA(16:0)	7.75	70.05	54.58	0.017*
FA(16:1)	7.52	71.12	67.12	0.143
FA(17:0)	7.87	5.96	5.92	0.431
FA(17:1)	7.67	4.14	4.12	0.102
FA(18:0)	8.02	121.58	118.3	0.223
FA(18:1)	7.8	211.56	200.59	0.087
FA(18:2)	7.6	394.16	279.8	0.010*
FA(18:3)	7.4	87.635	83.68	0.247
FA(18:4)	7.1	0.305	0.33	0.198
FA(20:0)	8.27	36.66	35.53	0.026*
FA(20:1)	8.05	15.66	15.24	0.265
FA(20:2)	7.86	0.95	0.94	0.487
FA(20:3)	7.67	0.55	0.56	0.272
FA(20:4)	7.55	0.04	0.05	0.082
FA(20:5)	7.47	0.03	0.03	0.280
FA(21:0)	8.39	0.16	0.15	0.074
FA(22:0)	8.51	1.2	1.19	0.431
FA(22:1)	8.28	6.17	5.92	0.041
FA(22:2)	8.09	0.27	0.27	0.823
FA(22:3)	7.92	0.048	0.05	0.535
FA(23:0)	8.62	0.04	0.04	0.785
FA(24:0)	8.75	3.17	3.05	0.146
FA(24:1)	8.51	1.34	1.21	0.258
FA(25:1)	8.84	0.03	0.02	0.327
FA(26:1)	8.96	1.27	1.2	0.026

Table S3. Top 7 enrichment KEGG pathways in the DGEs

Term	Name	GeneID
Fatty acid biosynthesis		
	acetyl-CoA carboxylase	LOC100164739
	fatty acid synthase	LOC100163154
	fatty acid synthase	LOC100158855
	fatty acid synthase	LOC100166618
	Very long-chain-fatty-acid-CoA ligase	LOC100164756
	fatty acid synthase	LOC100162692
	acetyl-CoA carboxylase	LOC100164739
Fatty acid metabolism		
	peroxisomal acylcoenzyme A oxidase	LOC100168090
	fatty acid synthase	LOC100163154
	fatty acid synthase	LOC100158855
	acetyl-CoA acetyltransferase	LOC100161259
	fatty acid synthase	LOC100166618
	Very long-chain-fatty-acid-CoA ligase	LOC100164756
	fatty acid synthase	LOC100162692
	Beta-ketoacyl synthase	LOC100166426
	fatty acid synthase	LOC100160920
	hypothetical protein V496_04806	LOC100150472
	Hybrid PKS-NRPS	LOC100167255
	stearoyl-coa desaturase-like	LOC100165835
	Palmitoyl-protein thioesterase 1	LOC100164869
	apolipoprotein D	LOC100168232
	long-chain-fatty-acid--CoA ligase 5	LOC100159821
	fatty acid desaturase-like	LOC100169386
	diacylglycerol o-acyltransferase-like	LOC100158996
	elongation of very long chain fatty acids protein 4	LOC100160501
Glycerophospholipid metabolism	acyl-sn-glycerol-3-phosphate acyltransferase delta	LOC100168710
	eye-specific diacylglycerol kinase	LOC100163751
	esterase FE4	LOC100164908
	diacylglycerol kinase theta	LOC100163089
	acetylcholinesterase	LOC100169250
	esterase FE4	LOC100166921
	acetylcholinesterase	LOC100168865
Drug metabolism cytochrome P450	UDP-glucuronosyltransferase 2B28	LOC100159463
	UDP-glucuronosyltransferase 2C1	LOC100164992

	UDP-glucuronosyltransferase 2B18	LOC100165933
	UDP-glucuronosyltransferase 2B2	LOC100166729
	carbonyl reductase [NADPH] 3	LOC100573774
	glutathione S-transferase 1-1-like	LOC100575419
Amino sugar and nucleo-tide sugar metabolism	beta-hexosaminidase subunit beta	LOC100163544
	uncharacterized	LOC100164767
	galactokinase	LOC100165228
	probable chitinase 10	LOC100169480
	beta-hexosaminidase subunit beta	LOC100572106
Phototransduction-fly		
	myosin 21	LOC100159321
	arrestin homolog	LOC100166316
Carotenoid biosynthesis		
	phytoene desaturase	LOC100159050
	phytoene desaturase	LOC100169110
	bifunctional lycopene cyclase /phytoene synthase	LOC100159332

Table S4. Transcriptome: up-regulated mRNAs (*Serratia*-free aphids vs *Serratia*-infected aphids)

ID	Log_2 FoldChange	P value	Description
LOC100163154	5.60709	1.16E-16	Fatty acid synthase
LOC100164739	1.33206	1.61E-11	Acetyl-CoA carboxylase
LOC100570731	1.43663	2.16E-10	
LOC103311179	2.49299	4.26E-07	
LOC100572794	1.58595	8.26E-07	
LOC100163544	2.64222	4.60E-05	Beta-hexosaminidase subunit beta
LOC100169480	1.48554	0.001591	Probable chitinase 10
LOC100572106	2.27238	0.000181	Beta-hexosaminidase subunit beta
LOC100575419	2.22623	0.040056	Glutathione S-transferase 1-1-like
LOC100166729	1.284047	0.018529	UDP-glucuronosyltransferase2B2
LOC100166921	1.176412	0.235230	Esterase FE4
LOC100169250	1.28845	0.090019	Acetylcholinesterase
LOC100158855	2.40127	0.001148	Fatty acid synthase
LOC100166618	2.25889	0.002162	Fatty acid synthase
LOC100164756	1.81140	0.014488	Very long-chain-fatty-acid-CoA ligase
LOC100166426	2.31567	3.87E-09	Beta-ketoacyl synthase
LOC100160920	1.73937	3.03E-06	Fatty acid synthase
LOC100150472	1.33882	5.95E-12	Hypothetical protein V496_04806
LOC100167255	2.43561	1.67E-17	Hybrid PKS-NRPS
LOC100165835	1.5757	0.002755	Stearoyl-coa desaturase-like
LOC100164869	2.7456	0.000308	Palmitoyl-protein thioesterase 1
LOC100168232	1.3883	5.97E-08	Apolipoprotein D
LOC100159821	1.2219	5.84E-24	Long-chain-fatty-acid-CoAligase5
LOC100169386	1.0187	0.007104	Fatty acid desaturase-like
LOC100158996	1.69396	4.37E-08	Diacylglycerol o-acyltransferase
LOC100160501	2.59936	0.001314	Elongation of very long chain fatty
LOC100158695	1.22726	0.000168	Acids protein 4
LOC100158721	3.20891	0.019663	Peroxidasin
LOC100159423	1.24383	0.017931	
LOC100159508	1.204384	0.012007	
LOC100159662	1.814033	1.814033	ABC transporter
LOC100159715	1.267733	0.001249	
LOC100160327	2.874810	0.046821	
LOC100160570	1.930340	0.016485	Protein toll
LOC100160667	1.000918	0.009778	
LOC100160931	2.219190	0.000109	
LOC100161055	1.145783	0.012816	
LOC100161211	1.464131	0.036032	
LOC100161957	2.144691	0.006669	

LOC100162011	1.603700	0.017726	Nucleosidetransporter 1
LOC100162036	1.556122	5.73E-05	
LOC100162607	4.881732	0.036356	
LOC100162732	2.383868	0.024785	Coagulation factor IX
LOC100163126	2.100723	2.100723	
LOC100163220	1.892318	0.015599	
LOC100163954	1.449654	0.020901	
LOC100163986	2.629901	1.48E-05	
LOC100164069	1.305396	0.003315	Serine protease
LOC100164120	1.172190	0.027381	
LOC100164275	3.198833	0.048365	
LOC100164310	1.813529	0.000661	Serine/threonine-protein kinase C
LOC100164750	1.47128	0.001180	
LOC100164752	1.962131	0.0001410.	Piwi-like protein
LOC100164783	1.684827	00012	
LOC100165095	2.258899	0.00459	
LOC100165417	1.159024	0.014488	
LOC100165476	1.576147	0.036445	Cystinosin homolog
LOC100165488	3.173848	0.020250	
LOC100165673	1.023242	0.004000	Neprilysin-2
LOC100166187	1.876333	0.024092	
LOC100166257	4.188113	0.000555	Farnesyl pyrophosphate synthase
LOC100166262	1.33696	0.000245	
LOC100166382	1.436634	0.009583	
LOC100166422	1.374283	1.91E-08	ATP2B4
LOC100166653	1.455633	0.000104	
LOC100166709	1.512762	2.06E-05	
LOC100166768	1.684178	0.004672	Lachesin
LOC100167094	2.099771	0.000312	
LOC100167407	1.171730	0.031154	
LOC100167607	1.412810	0.045475	
LOC100167758	1.111164	3.44E-10	
LOC100167851	1.369479	0.019102	Cuticle protein 21
LOC100168043	1.092281	0.000153	
LOC100168314	1.122146	0.016564	
LOC100168723	2.089085	0.025351	
LOC100169049	1.843082	0.004668	
LOC100169196	1.154489	1.71E-16	
LOC100169303	2.633608	0.045328	Glycine receptor alpha-3
LOC100169480	1.485542	2.45E-06	
LOC100568671	2.65239	0.001591	Probable chitinase 10
LOC100568743	1.42280	0.000440	
LOC100568800	2.574133	0.00019	

LOC100569220	4.782163	0.02051	
LOC100569970	1.041465	4.782163	
LOC100570058	3.631162	0.019575	Acidic phospholipase A2 PA4
LOC100570095	2.125009	1.02E-06	
LOC100570331	2.374829	1.60E-06	
LOC100570471	1.669547	0.000104	
LOC100570710	2.410412	0.001591	
LOC100570722	1.040924	0.043346	
LOC100570731	5.607099	1.15E-05	
LOC100570953	2.112082	1.16E-12	
LOC100571454	1.003363	0.004781	Copia protein
LOC100571876	3.267150	0.015055	Protein doublesex
LOC100571902	1.444230	0.000421	
LOC100572066	2.551028	0.001851	
LOC100572111	2.366745	0.013932	
LOC100572222	1.690026	0.030592	Cytokine receptor
LOC100572794	1.585955	0.032230	
LOC100572872	1.746319	4.71E-27	
LOC100573101	1.541512	1.746319	
LOC100573239	2.417439	0.000898	
LOC100573363	3.332764	0.049976	
LOC100573541	1.332064	0.009480	
LOC100574395	2.035154	1.61E-16	
LOC100574694	2.863365	0.035827	
LOC100574746	4.060835	0.00377	
LOC100575061	2.35861	0.031512	Low choriolytic enzyme
LOC100575228	2.1425	6.30E-05	
LOC100575313	2.94688	0.036389	
LOC100575442	2.22979	0.032259	
LOC100575453	1.23820	0.034644	Nucleoside transporter 1
LOC100575585	2.61954	2.09E-10	
LOC100575697	1.56348	5.00E-09	
LOC100576035	1.90937	1.563480	
LOC103307622	1.03651	0.027473	
LOC103308684	3.03123	0.005978	Putative nuclease HARBI1
LOC103311179	2.49299	0.001179	
LOC103311597	2.07913	9.26E-07	
LOC103311834	1.67475	0.025597	
LOC107884572	1.35375	0.017001	

Table S5. Transcriptome: down-regulated mRNAs (*Serratia*-free aphids vs *Serratia*-infected aphids)

Id	$Log_2FoldChange$	Pval	Description
LOC100159050	-2.11408	2.27E-07	Carotenoid biosynthesis
LOC100159332	-1.81659	0.003197	Lycopene cyclase
LOC100169110	-1.28092	8.43E-05	Phytoene desaturase
LOC100159321	-1.76809	9.39E-05	PREDICTED: myosin-1-like
LOC100166316	-1.9362	1.37E-05	Arrestin homolog
LOC100164767	-1.18396	8.22E-05	
LOC100165228	-1.25824	0.001329	Galactokinase
LOC100165933	-1.31714	3.29E-03	UDP-glucuronosyltransferase
LOC100164992	-1.40552	4.81E-09	UDP-glucuronosyltransferase
LOC100159463	-1.02464	3.07E-05	UDP-glucuronosyltransferase
LOC100168710	-1.-8679	0.00193	Acyl-sn-glycerol-3-phosphate
LOC100163751	-1.0489	0.000329	Eye-specific diacylglycerol kinase
LOC100164908	-2.4258	1.26E-09	Esterase FE4
LOC100163089	-1.37615	0.441849	Diacylglycerol kinase theta
LOC100168865	-1.14193	0.682173	Acetylcholinesterase
LOC100168090	-1.09993	0.000243	Acyl-coenzyme A oxidase 1
LOC100161259	-1.08225	7.71E-11	Acetyl-CoA acetyltransferase
LOC100162692	-1.124	0.00011	Fatty acid synthase
LOC100158692	-6.7620	0.019142	
LOC100158766	-1.146973	0.033684	
LOC100158864	-1.091578	0.0020584	Ninjurin-1
LOC100158867	-6.92246	0.0034648	Glucose dehydrogenase
LOC100158876	-1.275374	3.41E-07	Serine protease snake
LOC100159050	-2.114089	2.27E-07	
LOC100159160	-3.162287	0.004339	
LOC100159227	-1.22932	0.0100014	
LOC100159248	-1.563435	1.15E-09	Cytochrome P450 6k1
LOC100159332	-1.81659	0.0031976	
LOC100159394	-1.05927	2.60E-06	
LOC100159492	-1.36569	0.000201	Serine/threonine-protein kinase
LOC100159559	-1.34555	7.00E-06	Lysosomal alpha-mannosidase
LOC100159576	-2.675720	0.000138	Inositol oxygenase
LOC100159613	-2.288228	2.32E-05	
LOC100159632	-5.701265	0.00035	Glucose dehydrogenase
LOC100159743	-2.42333	0.007916	Aminopeptidase N
LOC100159883	-2.421970	9.01E-06	Histone H1E
LOC100159903	-1.086777	4.21E-06	
LOC100159959	-1.559299	3.61E-06	

LOC100159964	-2.532336	0.001229	Farnesol dehydrogenase
LOC100160046	-1.141177	1.24E-10	
LOC100160247	-1.040909	0.04822	
LOC100160265	-1.021612	0.04082	Asparagine synthetase
LOC100160300	-1.119753	3.97E-05	
LOC100160420	-1.855333	0.004648	
LOC100160582	-3.992376	0.006054	Acylphosphatase-2
LOC100160596	-1.021084	3.99E-05	
LOC100160601	-2.372834	0.010630	
LOC100160629	-1.605791	9.16E-05	Cytochrome P450 4C1
LOC100160644	-1.607102	0.03028	Protein takeout
LOC100160754	-1.573032	1.85E-15	Hydroxylysine kinase
LOC100160916	-1.023622	0.047174	
LOC100160991	-2.75621	0.045262	
LOC100160994	-1.44961	0.000298	Clavesin-1
LOC100161104	-1.706702	2.38E-10	
LOC100161219	-1.006278	0.003681	
LOC100161929	-5.02395	5.66E-05	
LOC100162471	-1.61100	0.0388638	Histone H1
LOC100162573	-1.519328	1.20E-07	
LOC100162704	-1.253468	0.004451	Sodium/nucleoside cotransporter
LOC100162745	-1.73478	0.008910	Histone H2A
LOC100162798	-1.122958	1.67E-06	
LOC100162817	-1.21998	0.013860	Triacylglycerol lipase
LOC100162846	-1.179335	4.25E-06	
LOC100162924	-1.253525	0.005138	
LOC100162973	-1.24519	1.67E-10	Clavesin-1
LOC100163035	-2.035881	1.54E-07	
LOC100163444	-1.094570	0.021609	
LOC100164189	-1.767213	0.000183	Growth/differentiation factor 8
LOC100164291	-1.882762	2.86E-05	
LOC100164454	-3.73108	0.004106	
LOC100164767	-1.183960	8.22E-05	Acidic mammalian chitinase
LOC100164880	-1.42381	0.009419	Regucalcin
LOC100164892	-1.093161	9.39E-06	NADPH-cytochrome reductase
LOC100165228	-1.25824	0.001329	Galactokinase
LOC100165676	-2.82030	0.033797	Hephaestin
LOC100165702	-1.00842	6.11E-05	
LOC100165822	-2.03483	3.51E-06	
LOC100165879	-1.85155	0.035197	
LOC100166467	-1.14744	0.014718	Glycine receptor subunit alpha-3
LOC100166509	-1.45807	0.046305	
LOC100166593	-2.55302	0.017331	Alpha-(1,6)-fucosyltransferase

LOC100166618	-1.28845	0.001148	
LOC100166638	-1.13545	1.32E-14	
LOC100166760	-2.13799	7.06E-06	Cytochrome P450 4C1
LOC100166927	-1.74212	0.0236232	
LOC100166970	-1.79771	2.48E-13	
LOC100167010	-1.27098	0.044246	
LOC100167028	-1.057136	0.012279	
LOC100167345	-1.28116	0.000402	
LOC100167427	-3.57503	0.003263	
LOC100167585	-2.22215	0.013218	Phenoloxidase-activating factor
LOC100167919	-4.62492	0.045018	
LOC100168023	-1.94998	0.021653	
LOC100168031	-1.45908	0.013019	Histone H2A
LOC100168063	-1.58957	0.0048788	
LOC100168111	-2.166415	0.034014	
LOC100168115	-1.11040	0.000772	
LOC100168229	-1.05069	0.013455	
LOC100168610	-1.41894	0.002499	P protein
LOC100168914	-2.91212	0.001871	
LOC100169110	-1.28092	8.43E-05	
LOC100169157	-1.05418	8.97E-05	
LOC100169218	-1.30138	0.009832	Alpha-(1,6)-fucosyltransferase
LOC100169243	-3.15466	2.16E-10	
LOC100169376	-1.80256	0.046749	
LOC100169576	-1.47552	0.000327	
LOC100169595	-2.74315	0.000183	Aminopeptidase N
LOC100169616	-1.25934	0.000850	Farnesol dehydrogenase
LOC100302376	-2.74484	0.013172	
LOC100302630	-1.86213	0.042031	
LOC100568563	-1.7695	0.016137	
LOC100568669	-1.47016	0.000807	Tetraspanin-9
LOC100568687	-1.17341	0.018892	
LOC100568834	-1.57929	0.019828	
LOC100568841	-1.40587	7.42E-06	
LOC100568871	-1.26546	0.000383	
LOC100569112	-1.69869	3.00E-08	
LOC100569135	-1.69896	8.21E-07	
LOC100569185	-1.16686	0.034217	
LOC100569360	-1.64581	0.004780	trehalose transporter Tret1
LOC100569372	-2.15774	0.022385	
LOC100569552	-1.46867	0.02182	
LOC100569593	-2.27974	0.001582	
LOC100569798	-1.02543	0.049276	

LOC100569981	-2.39273	0.031321	ABC transporter
LOC100570377	-1.15166	0.039760	
LOC100570454	-2.9998	0.014864	
LOC100570696	-2.1022	0.042998	
LOC100570714	-1.2911	0.000374	
LOC100570997	-1.3173	0.034562	
LOC100571147	-2.23157	0.019494	
LOC100571160	-1.42745	0.00874	
LOC100571239	-1.09996	0.027128	Ras-related protein Rab-10
LOC100571443	-1.11461	3.23E-08	
LOC100572030	-1.97254	0.005568	Histone H2A
LOC100572199	-1.32524	0.021702	
LOC100572425	-2.09741	1.17E-06	Glucose dehydrogenase
LOC100573179	-1.15217	0.007945	
LOC100573395	-2.28126	0.000698	
LOC100573463	-1.53557	0.019992	
LOC100573477	-1.746384	3.66E-07	
LOC100573638	-1.646761	0.009791	Aminopeptidase
LOC100573657	-1.046690	0.000198	
LOC100573697	-2.657097	0.000105	
LOC100573774	-1.296196	0.04389	Carbonyl reductase
LOC100573857	-3.093161	8.44E-05	Histone
LOC100573886	-2.014252	0.000341	
LOC100574043	-1.077503	0.047246	Probable ribonuclease ZC3H12C
LOC100574061	-1.272930	0.02028	
LOC100574449	-2.675044	0.00094	
LOC100574483	-3.30272	0.00058	
LOC100574507	2.057904	2.21E-18	
LOC100574511	-2.09362	0.029046	
LOC100574565	-3.17855	6.40E-07	
LOC100575108	-1.25858	0.049869	
LOC100575202	-3.579523	0.01534	
LOC100575435	-2.54618	0.016558	
LOC100575678	-1.79195	0.000334	
LOC100575704	-2.443701	0.043557	
LOC100575856	-1.03563	2.15E-05	
LOC103307796	-2.10277	1.44E-08	
LOC103308556	-1.02709	0.025373	
LOC103308614	-3.09806	0.049479	
LOC103308615	-2.04446	0.01518	
LOC103309375	-1.1401	0.000162	
LOC103309394	-2.72444	0.009512	
LOC103309625	-1.38700	2.50E-08	

LOC103309672	-1.73058	0.000133	
LOC103310130	-1.72090	9.21E-07	Zinc finger MYM-type protein 1
LOC103310416	-1.47229	0.045898	
151.3692022	-1.75549	0.006039	Histone H1
110.6518955	-1.09720	0.013966	Cubilin
101.7886722	-1.02698	0.003847	
11.29794195	-1.91815	0.029351	
12.00401616	-2.42177	0.008665	
LOC107882837	-1.26221	0.000344	Putative fatty acylCoA reductase
LOC107882937	-3.26779	0.027794	
LOC107882987	-1.65744	1.11E-05	
LOC107883167	-2.08122	0.041392	Cuticle protein 6
LOC107883317	-3.91614	0.006191	
LOC107883927	-1.41573	0.007397	
LOC107884415	-1.23253	0.028190	Glucose dehydrogenase
LOC115033297	-4.35067	0.003165	
LOC115033421	-1.00992	0.04556	
LOC115033768	-2.60558	0.01717	
LOC115034066	-2.65492	9.61E-21	Lactase-phlorizin hydrolase
LOC115034175	-2.03142	0.02535	
LOC115034434	-1.82807	0.00101	
LOC115034437	-1.14728	0.01584	
LOC115034452	-2.55499	0.01687	
LOC115034481	-1.88110	1.71E-09	
LOC115034970	-1.09550	0.012757	
NV22	-2.96264	0.041521	
rtv	-1.10794	0.00041	
LOC107882905	-2.47105	0.04689	
LOC107883010	-1.43266	0.521347	
LOC107883183	-2.50410	0.571981	
LOC107883841	-1.17528	0.16528	Zinc finger MYM-type protein 5
LOC107883980	-2.89046	0.045538	
LOC107884058	-1.22823	0.919242	

References

1. Anders S, Pyl PT and Huber W, HTSeq-a Python framework to work with high-throughput sequencing data. *Bioinformatics* **31**: 166-169 (2015).
